# Conversational Time Travel: Evidence of a Retrospective Bias in Real Life Conversations

**DOI:** 10.3389/fpsyg.2018.02160

**Published:** 2018-11-13

**Authors:** Burcu Demiray, Matthias R. Mehl, Mike Martin

**Affiliations:** ^1^Department of Psychology, University of Zurich, Zurich, Switzerland; ^2^University Research Priority Program “Dynamics of Healthy Aging”, University of Zurich, Zurich, Switzerland; ^3^Department of Psychology, University of Arizona, Tucson, AZ, United States

**Keywords:** Electronically Activated Recorder, mental time travel, autobiographical memory, future-oriented thought, retrospective bias, conversations, real life

## Abstract

We examined mental time travel reflected onto individuals’ utterances in real-life conversations using a naturalistic observation method: Electronically Activated Recorder (EAR, a portable audio recorder that periodically and unobtrusively records snippets of ambient sounds and speech). We introduced the term *conversational time travel* and examined, for the first time, how much individuals talked about their personal past versus personal future in real life. Study 1 included 9,010 sound files collected from 51 American adults who carried the EAR over 1 weekend and were recorded every 9 min for 50 s. Study 2 included 23,103 sound files from 33 young and 48 healthy older adults from Switzerland who carried the EAR for 4 days (2 weekdays and 1 weekend, counterbalanced). 30-s recordings occurred randomly throughout the day. We developed a new coding scheme for conversational time travel: We listened to all sound files and coded each file for whether the participant was talking or not. Those sound files that included participant speech were also coded in terms of their temporal focus (e.g., past, future, present, time-independent) and autobiographical nature (i.e., about the self, about others). We, first, validated our coding scheme using the text analysis tool, Linguistic Inquiry and Word Count. Next, we compared the percentages of past- and future-oriented utterances about the self (to tap onto conversational time travel). Results were consistent across all samples and showed that participants talked about their personal past two to three times as much as their personal future (i.e., retrospective bias). This is in contrast to research showing a prospective bias in thinking behavior, based on self-report and experience-sampling methods. Findings are discussed in relation to the social functions of recalling the personal past (e.g., sharing memories to bond with others, to update each other, to teach, to give advice) and to the directive functions of future-oriented thought (e.g., planning, decision making, goal setting that are more likely to happen privately in the mind). In sum, the retrospective bias in conversational time travel seems to be a functional and universal phenomenon across persons and across real-life situations.

## Introduction

Live in the here and now – so goes a common credo. However, one of the most remarkable skills of humans is not their ability to have their minds set on the present, but, rather, to engage in mental time travel. The human cognitive apparatus is a powerful time travel machine, allowing us to almost effortlessly project ourselves into the future to simulate possible future events, as well as put ourselves back into the past to relive our past experiences (Suddendorf and Corballis, 2007). Recently, psychologists have started to emphasize that memory (e.g., autobiographical memory) and prospection (e.g., future-oriented thought) are closely related phenomena that share many common qualities (e.g., Schacter et al., 2012; Klein, 2013; Rasmussen and Berntsen, 2013). Thinking or talking about our past and future are such natural, moment-to-moment activities that we do not notice or wonder how often we recall our memories or imagine our future in everyday life. Psychologists have started to explore this prevalence question using a range of self-report methods (e.g., diary method, experience-sampling) with a focus on participants’ thoughts. Here, we used, for the first time, an ecological behavioral observation method that is free of self-report to examine the prevalence of mental time travel behavior in everyday conversations (i.e., conversational time travel). Using the Electronically Activated Recorder (EAR; Mehl et al., 2001), we unobtrusively and intermittently sampled snippets of ambient sounds and speech from participants’ natural lives, and extracted information about their moment-to-moment conversations.

The first goal of the current research was to develop and validate a new, naturalistic observation approach to studying mental time travel reflected in everyday conversations. We listened to and coded participants’ recorded utterances in terms of whether they (a) had a time reference or not and (b) were about the self versus others. We tapped onto mental time travel by focusing on those utterances that were about the self with a time reference. In two studies, we validated our coding scheme using a text analysis program and with adult samples representing different age groups and countries.

The second goal of the current research was to examine how often people engage in conversational time travel, and when they do, how often they talk about their past versus future. There is some work on how much people *think* about their personal past versus future in everyday life (e.g., Klinger and Cox, 1987; Berntsen and Jacobsen, 2008; Gardner and Ascoli, 2015), but no work on how much they *talk* about their past versus future. The solitary nature of thinking versus the social nature of talking should have different effects on mental time travel (e.g., Kulkofsky et al., 2010), which has methodological and theoretical implications. Humans spend 32–75% of their waking time with other people (Mehl and Pennebaker, 2003). That is, much of human behavior occurs in a social context, therefore we examined, for the first time, mental time travel in the context of conversations. We unobtrusively observed and objectively coded the overt behavior of talking in everyday life to examine mental time travel reflected in people’s utterances.

### Prevalence of Past- and Future-Oriented Thoughts in Everyday Life

Previous studies measuring the incidence of subjective thoughts and experiences have typically used variants of the original experience-sampling method (ESM; Csikszentmihalyi et al., 1977). One type of ESM is *event-contingent sampling* (e.g., diary method; Berntsen, 2007) in which diary entries are prompted by participants through introspection and detection of the occurrence of a target event. The second type is *signal-contingent sampling*, which requires people to evaluate the presence of a targeted experience when prompted by a randomly timed signal (e.g., Pasupathi and Carstensen, 2003). One important advantage of these methods is their high ecological validity.

Using the diary method, D’Argembeau et al. (2011) explored the frequency of thinking about the personal future in everyday life. They asked participants to report whenever they realized that they were thinking about their future and found that participants reported experiencing, on average, 59 future-oriented thoughts on a typical day. In contrast, Rasmussen and colleagues (i.e., Rasmussen and Berntsen, 2011; Rasmussen et al., 2015) examined the frequency of thinking about the personal past (i.e., autobiographical memories) using the diary method. They made a distinction between voluntarily thinking about memories versus involuntary memories (which spontaneously pop up without deliberate search) and compared their frequency. They showed that participants self-reported recalling on average 7–8 voluntary and 20–22 involuntary autobiographical memories per day. Taken together, these two studies suggest that young adults think about their personal future twice as much as their past. Berntsen and colleagues (Berntsen and Jacobsen, 2008; Finnbogadottir and Berntsen, 2013), however, did not replicate this finding. They used the diary method to examine involuntary mental time travel and compared the frequency of involuntary memories and involuntary future-oriented thoughts. They found that involuntary memories were as frequent as involuntary future-oriented thoughts in daily life (around 22 per day).

In a signal-contingent experience sampling study, Gardner et al. (2012) examined the frequency of thinking about the personal past (i.e., autobiographical memories) in everyday life. Via random prompts throughout the day, they asked young adults to report whether they were thinking about a specific autobiographical memory at that moment or not. They found that the probability of being caught while recalling a specific autobiographical memory was 15%. However, in a second study using the same method, Gardner and Ascoli (2015) found this probability to be 10%. It was unclear to the authors why this small discrepancy occurred, but they suggested it might be due to the investigation of both past- and future-oriented thoughts in the second study. They found that participants thought about their future about 13% of the time. The second study’s results are in line with an early signal-contingent experience sampling study: Klinger and Cox (1987) have shown that people rated 12% of their momentary thoughts as focused on their past and 12% on their future (versus 67% on their present). However, Felsman et al. (2017) found a large difference between past- and future-oriented thoughts. Via text messages throughout the day, they asked participants to report which of the following would best characterize their thoughts: past-, present- or future-focused. They found that people reported focusing much more on the future (26%) than the past (8%), with present as the most frequent category (66%).

Signal-contingent experience sampling has been used to examine involuntary thoughts, as well, particularly mind-wandering. Mind-wandering is defined as a shift of attention from a primary task in the present toward internal information or self-generated thought, such as autobiographical memories (Smallwood and Schooler, 2006). Song and Wang (2012) examined the temporal orientation of mind wandering by randomly prompting participants throughout the day and asking whether they were mind wandering or not, and if mind wandering, whether they were thinking about past, future, present or atemporal events. They found a *prospective bias* such that participants were mind wandering about the future (40.53%) twice as much as the past (21.53%). We should note that this prospective bias has been repeatedly shown in laboratory studies of mind wandering (e.g., Smallwood et al., 2009; Baird et al., 2011; Stawarczyk et al., 2011). However, researchers have identified some factors that affect the temporal orientation of mind wandering, with some eliminating the prospective bias, such as manipulating the experimental settings (e.g., response options and cues; Jackson et al., 2013; Vannucci et al., 2017), and controlling for participant characteristics such as familiarity with the task (Smallwood et al., 2009) and mood (Poerio et al., 2013).

In sum, all studies reviewed above are conducted in the real world, focused on thoughts (retrieved voluntarily and/or involuntarily) and based on self-report (i.e., diary method and signal-contingent experience sampling). They have resulted in two different findings on the prevalence of thinking about the personal past versus future: Some reported that future-oriented thoughts occur almost twice as frequently as past-oriented thoughts, whereas others reported similar proportions of both.

### Temporal Orientation

Another line of research that is relevant for our work is time perspective or temporal orientation. Temporal orientation refers to relatively stable individual differences in the relative emphasis one places on the past, present, or future (Zimbardo and Boyd, 1999). Temporal orientation has been widely examined in relation to personality traits (e.g., Zhang and Howell, 2011), academic outcomes (e.g., Horstmanshof and Zimitat, 2007), risky behaviors (e.g., Daughterty and Brase, 2010), and health outcomes (see Stolarski et al., 2015 for reviews). Temporal orientation is usually assessed with surveys, such as the Zimbardo Time Preference Inventory (ZPTI; Zimbardo and Boyd, 1999) and Balanced Time Perspective Scale (BTPS; Webster, 2011). Jason et al. (1989) interviewed women (mean age = 31) and asked them to rank their past, present and future by the amount of thinking time devoted to each temporal focus. Women self-reported thinking about their present 41% of the time, their future 38% of the time and their past 21% of the time. This finding is similar to others (reviewed above) showing that future-oriented thoughts occur twice as much as past-oriented thoughts.

There is only one study on temporal orientation that is not based on self-report: Park et al. (2016) have created a novel language-based measure of temporal orientation: They have developed a model to automatically classify individuals’ social media messages as oriented toward the past, present or future (model accuracy = 72%). They used the model to classify over 1.3 million Facebook status updates (i.e., short text messages) written by 5,372 individuals aged 13–48. They found that 65% of messages were present-oriented, 19% were past-oriented and 16% were future-oriented. This result does not fit with the questionnaire findings above and presents an equal proportion of past- and future-oriented messages.

In conclusion, studies based on self-report (i.e., diary method, signal-contingent experience sampling, questionnaires) and one study based on a linguistic analysis of social media messages (Park et al., 2016) have resulted in two findings: a prospective bias versus an equal proportion of past- and future-oriented thoughts. All of these studies have focused on thoughts, therefore, we can conclude that future-oriented thoughts tend to dominate our private mental worlds compared to past-oriented thoughts.

In contrast, our social worlds might be dominated by past-oriented thoughts: Humans spend one fifth of their waking time in spontaneous conversation (Dunbar, 1998) and a significant portion of this time is dedicated to talking about past events (Eggins and Slade, 1997; Dessalles, 2018). According to Desalles (2007), the function of recalling the past is to accumulate stories that are relevant to tell in conversation. He claims that events that are memorable are exactly those that are good for narrating. Similarly, Mahr and Csibra (2018) argue that the main function of remembering is communication. They claim that social interactions require the justification of entitlements and obligations, which is possible only by reference to past events. In sum, these theoretical accounts highlight the importance of recalling the personal past in conversations. Therefore, we examined, for the first time, mental time travel in conversations and explored whether there is a *retrospective bias* in talking behavior, in contrast to the prospective bias observed in thinking behavior (e.g., Felsman et al., 2017).

### Overview of the Present Studies

The most important novelty of this work is its naturalistic observation approach to studying spontaneous, everyday conversations unobtrusively and with minimal participant burden. We used the Electronically Activated Recorder in both studies to collect random snippets of everyday conversations. The EAR is a portable audio recorder that intermittently records brief snippets of ambient sound and speech (Mehl et al., 2001). It captures acoustically detectible aspects of participants’ environments, such as their locations, activities and social interactions (Mehl, 2017). The strength of the current work is its attempt to increase ecological validity through sampling from a wide range of natural situations: We obtained a huge sample size by collecting more than 32,000 sound snippets.

The EAR has been used with good acceptance and compliance (Mehl, 2017), in all age groups (Bollich et al., 2016; Demiray et al., 2017) with healthy and clinical populations (e.g., Robbins et al., 2014). The psychometric properties of EAR-observed conversational behavior have been established in prior research with student (Mehl and Pennebaker, 2003) and adult populations (Bollich et al., 2016). Study 1 has been approved by the Institutional Review Board of the University of Arizona, and Study 2 was approved by the Ethics Committee of the University of Zurich. We have implemented a series of safeguards to protect participants’ privacy and to ensure data confidentiality. First, the EAR recorded only a small fraction of the day (e.g., 2.5% when sampling 30 s). Second, participants had the opportunity to review their recordings and erase any files they did not want on record, before the investigators accessed the data. Third, in order to protect bystanders, we encouraged participants to wear the EAR visibly (with large warning stickers on them) and to readily mention the study to others. Finally, although sound files included bystanders’ utterances, we only coded and analyzed the utterances of our participants (for a detailed discussion of EAR privacy and confidentiality policies, see Mehl, 2017; Robbins, 2017).

In order to examine mental time travel as reflected in participants’ utterances, we developed a novel coding scheme: We, first, coded whether participants’ utterances were time-dependent (i.e., had a reference to time) or time-independent (e.g., semantic memory such as “The name of the restaurant is Satchel’s”; Suddendorf et al., 2009). Next, we coded whether time-dependent utterances were about the self (i.e., autobiographical) or about others (e.g., vicarious memories; Pillemer et al., 2015). Finally, in order to tap onto mental time travel, we focused on the autobiographical, time-dependent utterances: We coded for “personal past” when the participant was talking about personally experienced past events (e.g., “I visited my grandparents last week”). “Personal future” was about anything that will/might or not happen in one’s future (e.g., “Next year I’m starting my MA degree”). Finally, when the participant was talking about their current activity, task or situation, we coded for “present” (e.g., “This show is boring, let’s change the channel”).

In Study 1, we validated our coding scheme using the Linguistic Inquiry and Word Count (LIWC), which is currently the most extensively validated text analysis tool in the social sciences (LIWC; Pennebaker et al., 2007). In Study 2, we validated the coding scheme with different samples. Previous studies on mental time travel have mostly focused on (1) college students or young adults, (2) one culture, with no cross-cultural comparisons, (3) experiences of a single temporal focus, such as only autobiographical memories or only future-oriented thoughts (e.g., Pasupathi and Carstensen, 2003; Mace, 2004; Kvavilashvili and Fisher, 2007; Schlagman and Kvavilashvili, 2008; Schlagman et al., 2009; D’Argembeau et al., 2011; Rasmussen and Berntsen, 2011; Gardner et al., 2012). Important and novel aspects of the current work is the inclusion of (1) participants that represent the whole adult life span, (2) participants from two countries, and (3) both past- and future-oriented utterances. We compared the prevalence of past- and future-oriented utterances across young, middle-aged and older adults in the United States and Switzerland. Study 1 examined the utterances of healthy spouses of breast cancer patients over a weekend (United States), and Study 2 examined the utterances of healthy young and older adults over 4 days (Switzerland). In addition to sampling such a wide range of individuals, one novel achievement of this work is its sampling from the universe of real-life situations.

## Study 1

This study is part of a larger project on American couples coping with breast cancer. Breast cancer patients and their healthy spouses were recruited at the Arizona Cancer Center, as described in earlier work that examined cancer conversations of couples (Robbins et al., 2014). For the purposes of our research, we focused only on the spouses’ utterances. The reason we used this dataset is that it was the only readily available dataset with Ear transcripts that we could use to develop our coding scheme.

The first goal of Study 1 was to validate our coding scheme using the LIWC (Pennebaker et al., 2007). We used LIWC to count specific words in participants’ utterances. We first compared utterances manually coded as time-dependent and those coded as time-independent in terms of the following LIWC variables: future-tense and past-tense. We expected time-dependent utterances to include significantly more verbs with tense than time-independent utterances. Second, we compared autobiographical (self-related) and others-related utterances in terms of personal pronouns: We expected self-related utterances to include more 1st person singular and plural pronouns, whereas others-related utterances to include more 2nd and 3rd person pronouns. Finally, utterances coded as personal past, personal future and present were compared in terms of their verb tense. We expected, for example, utterances about the personal past to include more verbs with the past tense than utterances about the present and personal future.

The second goal of Study 1 was to examine the prevalence of mental time travel in participants’ utterances, and to specifically compare the frequency of past- versus future-oriented utterances. Recent theories on episodic memory (Desalles, 2007; Mahr and Csibra, 2018) suggest that the main function of remembering the past is communication. Past research on autobiographical memory emphasizes significant social functions of memories showing that people recall their personal past to provide material for conversation (Pasupathi et al., 2002), to update others about what is ongoing in their life (Webster, 2003), to create/enhance intimacy in relationships (Alea and Bluck, 2007), to elicit empathy for others (Bluck et al., 2013) and to teach and inform others (O’Rourke et al., 2017). In contrast, future-oriented thinking is shown to serve directive functions such as planning, decision making, problem solving, goal intention and goal achievement (e.g., Szpunar, 2010; D’Argembeau et al., 2011; Schacter et al., 2017). Such directive functions should be inherently private and more likely to occur when people are thinking alone (O’Rourke et al., 2017). For example, Kulkofsky et al. (2010) have shown that private reminiscence favors directive functions (which guide current and future behavior), whereas social contexts are associated with memories that have higher social functions. Thus, we expected to observe significantly more autobiographical memories (i.e., past-oriented utterances) than future-oriented utterances in the social setting of conversations with others. That is, we expected a *retrospective bias* in conversational time travel in contrast to the prospective bias observed in mental time travel (e.g., Jason et al., 1989; Song and Wang, 2012).

### Materials and Methods

#### Sample

Our sample of real-life situations included 9,010 sound snippets collected from 51 healthy spouses. Out of 51 spouses, 44 were male (86%). Participants were on average 59 years old (Range: 26–94, *SD* = 14). Eighty-two percent of participants were Caucasian (n = 42), 15% Latin American (n = 8), and 2% Asian (n = 1). All participants were in a marriage-like relationship, and were primarily English speaking. Each couple received $150 for their participation.

#### Procedure

The first study session usually occurred on a Friday afternoon. All participants, first, gave written informed consent in accordance with the Declaration of Helsinki. They, then, completed a set of questionnaires as part of the larger study, and were provided with an introduction to the EAR. They were told that the device should be worn as much as possible over the weekend during their waking hours. They were informed that the EAR would record 50 s of ambient sound at a time for a total of approximately 10% of their waking hours. Participants were informed that the snippets would be recorded without their awareness and they should proceed with their normal, everyday life as much as possible. They were also told the EAR would cease recording during sleeping hours. All participants were explicitly told they would have an opportunity to review all recordings prior to anyone listening to them and to erase any files they did not want on record. Following that weekend, typically on the Monday, the EAR devices were collected from the participants and another battery of questionnaires, which included demographics and medical information, was administered. Participants were debriefed and given a password-protected Cd containing all of their sound files to review. There were over 9,000 sound files collected and of those only one participant deleted just one file.

#### Measures

The EAR was software programed on an Hp iPaq 100 handheld computer. The device was set to record 50 s every 9 min. This sampling rate has been established in previous studies as yielding stable estimates of habitual daily behavior (Mehl et al., 2012). The device was housed in a protective case affixed to participants’ waistlines, and an external microphone (Olympus Me-15) was attached to participants’ lapels. The EAR was preprogrammed to not record for 6 h during the participants’ predefined normal sleep hours, starting 30 min after they indicated they typically go to sleep. The EAR recorded participants’ waking days, from the time the participant received the device until they went to sleep on Sunday. This yielded an average of 176 (Sd = 57) valid, waking sound files (approximately 2.4 h of data per participant), which was defined as a file where the participant was wearing the Ear with no technical difficulties, while the participant was awake.

##### EAR-Derived measures: coding of sound files

All sound files were listened to, transcribed and coded by trained coders. Files were coded, as part of the larger project, for whether the participant was talking or not. For the goals of the current study, we developed a coding scheme for the temporal focus of participants’ utterances (See Table 1 for examples, and note that we make all coding guidelines available upon request to interested researchers). We first differentiated between time-dependent versus time-independent utterances. *Time-independent utterances* had no reference to time and included semantic memory (i.e., general knowledge about the world, such as “Paris is the capital of France”) and personal comments, beliefs, preferences, attitudes about anything in general (e.g., “He’s really nice”). *Time-dependent utterances* included a reference to time (i.e., past, present, and/or future) and were divided into autobiographical (self-related) and others-related categories. Autobiographical utterances were about the personal past, present moment and personal future, whereas utterances about others focused on others’ past and others’ future. *Personal past* refers to talking about personally experienced past events: These could be specific events (that happened at a particular place and time), repeated events (e.g., “I used to go to the gym every day”), extended events (e.g., “our 2-week vacation last Christmas”), and long periods of life (e.g., “When I lived in the United States”; Conway et al., 2004). In contrast, *others’ past* refers to talking about other people’s past experiences (i.e., the participant did not experience the event himself/herself). *Personal future* refers to anything that will/might or will/might not happen in one’s future (e.g., “We will not go to the movies”). *Others’ future* refers to talking about other people’s future experiences, which the participant is not personally involved in (e.g., “They might go skiing next week”). Finally, utterances about the *present* refer to talking about the current activity, task or situation. This also includes extremely recent past and extremely close future, which is connected to the present moment (e.g., “I just washed the potatoes and I am going to cook the veggies now”). There is no “others’ present” category, as the participant has to be there to observe others’ present activity, which automatically involves the participant’s present. Utterances such as “David is at the cinema” were coded as “time-independent,” as semantic knowledge.

**Table 1:** Examples of each coding category.

Time-independent:	Personal comments, attitudes, etc.	Semantic memory
	“Yeah, I like her. She comes off as, umm, a very unassuming person.”	“There’s always more of the other types of apples than there are Gala. You can buy Delicious for ninety nine cents a pound.”
	“If he just wasn’t so arrogant and such a know it all, I mean to me that’s a red flag because of Xxxx. Somebody that says they know it all, that they’re smarter than all the teachers. That’s a run for your life kind of thing because I always think of what’s his name. That’s not a good thing. The world is so much bigger than a restaurant.”	“Some of these guys look younger than the others. 36 years these guys have been playing mariachi. They’ve been playing at Epcot since 82. So Florida is now their home.”

**Time-dependent:**	**Autobiographical (Self)**	**Others**

Past	“Well what happened, a truck went by us really really fast. A big red truck. No, but he passed us no more than this far away and then all of the sudden boom. So what happened was, somebody was chasing him and hit us.”	“When he talked to her about it though, was he nice to her about it?”


		“She finally said, ok I’ll take it. And she was mad, she was mad at the world. She made herself sick, she was madder than hell. So she took off. And living in a, uh, she’s living in someplace 800 square feet.”
Future	“Well, this won’t take 10 min and then we’ll go get the blue car. We can get employment application forms at the stationary store, can’t we?”	“xxxx is going to be thirteen next week and xxxx is eleven. Xxxx goes to Dulin in the gate program and xxxx’s finishing up at Sam Huges this week, or next week. And xxxx?”
Present	“Okay. Now do you want to go by Albertson’s first just to see if they’re still open? It’s just right across the street. If it were really out of the way I would suggest that we not do it but. Yes. Do you want your sunglasses on honey? Pull in over here though, so you don’t block traffic.”	


*“Xxx” is for the de-identification of individuals.*

All coding categories were dichotomous, indicating presence (1) or absence (0) of a temporal focus. In addition, each sound file was coded in a TIME column with 1 = personal past, 2 = others’ past, 3 = present, 4 = personal future, 5 = others’ future, 6 = time-independent (See Table 2 for examples). Categories were not mutually exclusive, such that any 50-s sound file might include any combination of temporal foci. For example, if one talked about both personal past and others’ past within the same sound file, they received a 1 for both temporal categories and a “1–2” for the TIME variable. Each sound file was double-coded by two coders. We calculated inter-rater reliability by using the TIME variable, but not the single temporal focus variables separately: The two coders agreed on the TIME variable 64.12 % of the time. This calculation of inter-rater reliability was much stricter than calculating inter-rater reliability for each temporal focus separately: It is less likely to obtain agreement in the TIME variable, especially in specific cases such as a coding of “1–3–4,” than obtaining agreement separately in single columns (e.g., separately for 1 = personal past, 3 = present, 4 = personal future). Nevertheless, all sound files that showed a disagreement between the two coders were re-listened to and the disagreement was resolved through discussion.

**Table 2:** Examples of the coding scheme.

	TIME	Personal past (1)	Other’s past (2)	Present (3)	Personal future (4)	Others’ future (5)	Time-independent (6)	Dominant time
There it is. So then, it’s trash? What’s this? I found this in an envelope. What is this? Well, it was in an envelope. I’m going to give it to my mom. OK. That goes with this and this. These two go together.	1-3-4	1	0	1	1	0	0	3
I went to the gym yesterday.	1	1	0	0	0	0	0	1


*DOMINANT TIME exists in only Study 2. All variables except for TIME and DOMINANT TIME are dichotomous variables.*

##### Text analyses

Transcriptions of utterances were analyzed using LIWC (Pennebaker et al., 2007). LIWC software is one of the most widely used and best-validated text analysis tool in psychological science (e.g., Pennebaker et al., 2003; Tausczik and Pennebaker, 2010). LIWC analyzes text word-by-word and categorizes it into different linguistic (e.g., pronouns, prepositions) and psychological categories (e.g., emotion words, social words). It creates a percentage of word use (specific category/total number of words) by categories for each participant. In the current study, we used the following categories: past-tense, future-tense, present-tense and all personal pronouns.

### Results

A total of 4,100 sound files included participant speech (45.5% of valid sound files). We were unable to code for temporal focus in 747 sound files (18%) due to the brevity or vagueness of speech. The average number of words in these transcripts was four (e.g., “The what? Oh yes,” “Me, um, I guess”) and there were many cases with information that could help identify participants (e.g., names). We excluded 115 sound files (3.4 %) that were related to cancer in order to examine only ordinary daily conversations. Analyses were conducted with the remaining 3,238 sound files: In order to run the following analyses of variance, this dataset with one sound file on each row (sound-level dataset) was converted into a person-level dataset (one row is one participant) which aggregated data on the person level. Note that we make all data available upon request to interested researchers.

#### Validation of the Coding Scheme

The first goal of Study 1 was to validate our coding scheme using the Linguistic Inquiry Word Count (Pennebaker et al., 2007). Participants’ verbatim EAR transcripts were submitted to LIWC. We first compared utterances manually coded as time-dependent and those coded as time-independent in terms of their verbs with past-tense and future-tense. We conducted a repeated-measures MANOVA and found that time-dependent utterances included significantly more verbs with past tense (*M* = 4.58, *SD* = 1.38) than time-independent utterances (*M* = 2.62, *SD* = 2.19); *F*(1,45) = 29.64, *p* < 0.001, $\eta_{p}^{2}$ = 0.40. Similarly, they included significantly more verbs with future tense (*M* = 2.18, *SD* = 0.68) than time-independent utterances (*M* = 0.78, *SD* = 0.99), *F*(1,45) = 62.41, *p* < 0.001, $\eta_{p}^{2}$ = 0.58. That is, utterances that we had coded as time-dependent included more verbs with past and future tense than utterances coded as time-independent, which validated our coding.

Second, we compared autobiographical (self-related) and others-related utterances in terms of the number of their personal pronouns. We aggregated the number of pronouns on the person level, conducted a repeated-measures MANOVA and confirmed our expectations: We found that self-related utterances included significantly more 1st person singular pronouns (*M* = 5.29, *SD* = 1.42; *F*(1,43) = 132.58, *p* < 0.001, $\eta_{p}^{2}$ = 0.76) and 1st person plural pronouns (*M* = 1.22, *SD* = 0.75) than others-related utterances (singular: *M* = 1.19, *SD* = 1.89; plural: *M* = 0.12, *SD* = 0.45), *F*(1,43) = 75.31, *p* < 0.001, $\eta_{p}^{2}$ = 0.64. In addition, we found that the number of 2nd person pronouns (*M* = 5.85, *SD* = 4.98), 3rd person singular pronouns (*M* = 3.64, *SD* = 3.73), and 3rd person plural pronouns (*M* = 2.01, *SD* = 3.27) in others-related utterances was significantly higher than the number of 2nd person pronouns (*M* = 3.60, *SD* = 1.31), 3rd person singular pronouns (*M* = 1.41, *SD* = 1.01), and 3rd person plural pronouns (*M* = 1.01, *SD* = 0.55) in self-related utterances, *F*s(1,43) ranged 4.21–8.32, $\eta_{p}^{2}$ ranged 0.09–0.29, *p*s < 0.05. That is, utterances that we had coded as autobiographical were more about the self with pronouns such as “I,” “me,” “we,” and “us,” whereas others-related utterances were more about second and third persons (e.g., “you,” “he,” “she,” “they,” and “him”).

Finally, we validated our conversational time travel coding by comparing utterances manually coded as personal past, personal future and present in terms of their verb tense. We conducted a repeated-measures MANOVA and found, as expected, that utterances coded as personal past included a significantly higher number of verbs with past tense (*M* = 8.39, *SD* = 2.61) than utterances coded as personal future (*M* = 0.88, *SD* = 0.98) and present (*M* = 1.83, *SD* = 0.90), *F*(1,39) = 294.72, *p* < 0.001, $\eta_{p}^{2}$ = 0.88. In contrast, we found that utterances coded as personal future included a significantly higher number of verbs with future tense (*M* = 2.48, *SD* = 2.06) than utterances coded as personal past (*M* = 0.66, *SD* = 0.75) and present (*M* = 0.69, *SD* = 0.48), *F*(1,39) = 28.25, *p* < 0.001, $\eta_{p}^{2}$ = 0.42. Finally, we confirmed that utterances coded as present included a significantly higher number of verbs with present tense (*M* = 15.62, *SD* = 2.41) than utterances coded as personal past (*M* = 8.60, *SD* = 2.62) and personal future (*M* = 13.92, *SD* = 4.76), *F*(1,39) = 34.47, *p* < 0.001, $\eta_{p}^{2}$ = 0.47. In sum, all of our expectations regarding our coding categories were confirmed and we succeeded in validating the coding scheme with LIWC.

#### Frequency of Past- Versus Future-Oriented Utterances

The second goal of Study 1 was to examine the prevalence of mental time travel in participants’ utterances, and to compare the frequency of past- versus future-oriented utterances. In order to calculate percentages, we used the sound files that included only a single temporal category (e.g., only personal past, only present or only future) and excluded those that involved more than one temporal focus (e.g., sound file that includes both personal past and personal future). This allowed us to take “sound file” as the unit of analysis and use those sound files that had a single temporal category to clearly count the frequencies of purely past- versus future-oriented sound files.

**FIGURE 1:**
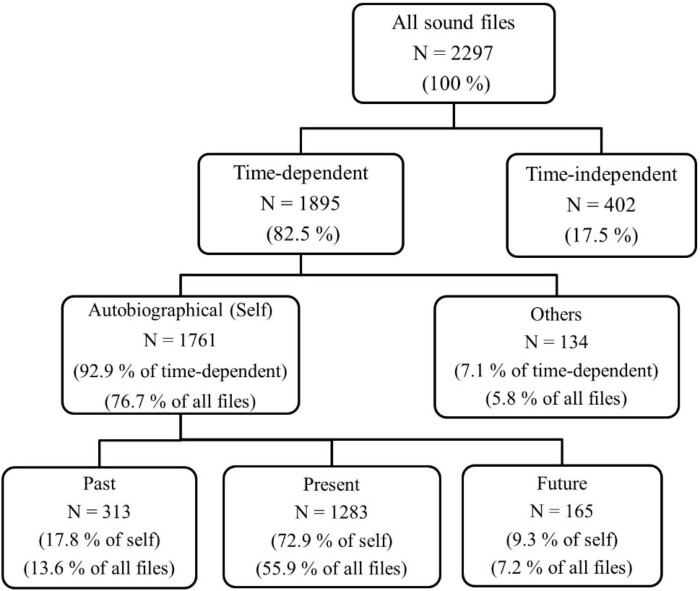
Study 1: frequencies and percentages for each temporal category. All sound files (100%) refers to all sound files that include speech with no technical problems.

There were 2,297 sound files that included only one temporal category (Figure 1, top row). Out of these, 17.5% were time-independent and included utterances presenting semantic memory or personal preferences, ideas and beliefs (Figure 1, second row). Out of the sound files that were time-dependent, 93% were about the self and 7% were about other people (Figure 1, third row). Utterances about others were further categorized as others’ past (*N* = 92, 68.7%) and others’ future (*N* = 42, 31.3%). Sound files that included self-related utterances were further divided into past (17.8%), present (72.9%), and future categories (9.3%) to present mental time travel (Figure 1, bottom row). That is, participants talked about their personal past in 13.6% of all their sound files and about their future in 7.2% (This means they engaged in conversational time travel in 20.8% of their sound files).

We ran a repeated-measures ANOVA to compare the number of past-, present-, and future-oriented utterances. For this analysis, we used the aggregate person-level amount of talking about the past, present versus future. We found that people talked significantly more about their past (*M* = 6.08, *SD* = 4.53) than their future (*M* = 3.17, *SD* = 2.60), *t*(51) = -5.47, *p* < 0.001. Furthermore, pairwise comparisons showed that present-oriented utterances (*M* = 24.71, *SD* = 14.51) were significantly more frequent than both past- and future-oriented utterances, *F*(2,50) = 78.69, *p* < 0.001, η_ρ_^2^ **=** 0.76.

### Discussion

We observed, over a weekend, the daily conversations of healthy spouses of breast cancer patients and developed a coding scheme for the temporal focus of their utterances. The first goal of the study was to validate our coding scheme using a text analysis tool: We succeeded and showed that utterances manually coded as (1) time-dependent versus time-independent, (2) self-related versus others-related, and (3) past-, present- versus future-oriented were indeed different from each other in terms of the words they included.

The second goal of the study was to explore the prevalence of conversational time travel in everyday life and to compare the frequency of past- and future-oriented utterances. Our coding scheme first revealed that individuals mostly produced time-dependent utterances (82.5% of all sound files). Semantic information and general comments about the world occurred in only 17.5% of the sound files. Second, we found that individuals talked in a self-referential way most of the time: 77% of all sound files and 93% of time-dependent sound files included autobiographical utterances. In contrast, participants talked about other people in only 5.8% of the sound files. This suggests that vicarious memories (Pillemer et al., 2015) and vicarious future-oriented utterances (e.g., Grysman et al., 2013) are quite rare in daily conversations. This is the first study to examine the prevalence of vicarious thoughts about others and to explore them in everyday life, therefore these findings may inspire future work.

Finally, we examined mental time travel as reflected in autobiographical utterances and found that 13.6% of sound files were about the personal past, whereas 7.2% were about the personal future. That is, people talked about their personal past almost twice as much as their personal future, and the difference was significant. This is in line with our expectation of a retrospective bias in the social setting of conversations (e.g., Eggins and Slade, 1997). Participants referred to their past much more than their imagined future while interacting with others. This is in contrast to previous work on private thoughts: While thinking, people seem to focus more on the future than the past (e.g., Andrews-Hanna et al., 2010) or focus equally on both (e.g., Klinger and Cox, 1987; Gardner and Ascoli, 2015). One explanation might be that recalling past events (i.e., autobiographical memories) may be more useful than simulating future events in social interactions (e.g., Desalles, 2007). We know that talking about memories serves social functions such as creating/enhancing feelings of intimacy, feeling empathy toward others, creating/enhancing conversation, teaching and giving advice (e.g., Alea and Bluck, 2003; Webster, 2003; O’Rourke et al., 2011, 2013, 2017). In contrast, prospection may be more functional while thinking, as private thoughts tend to serve higher directive functions such as setting goals, planning and decision making (e.g., Kulkofsky et al., 2010; Szpunar, 2010; D’Argembeau et al., 2011). For example, Rasmussen and Berntsen (2013) asked participants in the laboratory to remember two events from their past and to imagine two events from their future, and to rate each event on their perceived functions. Past events were rated higher than future events on the social function, as well as on their frequency of being shared with others. Cole et al. (2016) also asked participants to recall past events and imagine future events using a laboratory paradigm and found that people reported *thinking* about future events more often than past events. In sum, we believe that the social nature of conversations creates an efficient context for memories to be recalled in everyday life.

Present was the most frequent category, with 60% of all sound files being about the current activity or situation. This shows that while people are talking, more than half of their utterances are focused on what they are actually doing or observing (i.e., goal pursuit, Klinger, 2013). This is in line with previous work: In two experience-sampling studies, individuals rated 66% and 67% of their momentary thoughts as focused on the present (Klinger and Cox, 1987; Felsman et al., 2017, respectively). Similarly, Park et al. (2016) showed that 65% of participants’ social media messages were present-oriented. In sum, present orientation is found to occupy about 60–67% of both our thoughts and utterances, as assessed with three different methodologies.

Study 1 had some limitations. The sample included partners of cancer patients. This may have biased the situation samples toward a present- or past-orientation. However, only 3.4% of situation samples included conversations about the cancer, which we eliminated from our analyses, therefore we assume that there should be a minimal bias. Still, it is an open question to which degree the situation samples would differ with a population that is not associated with cancer. Furthermore, most of the participants were men and middle-aged. Therefore, in Study 2, we tried to obtain more gender-balanced samples from both young and late adulthood. A second limitation was that sound files were collected over a weekend. Although 2 days of EAR sampling has proven to yield reliable data (e.g., Mehl and Pennebaker, 2003), it is important to show that our findings are not an artifact of sampling situations insufficiently or sampling situations over a weekend. Therefore, in Study 2, we collected data across 1 weekend and 2 weekdays, with a counterbalanced order. Another limitation was that our inter-rater reliability calculation was overly strict, which led to a lower agreement between coders than expected. In Study 2, we used the same strategy for consistency across studies, but also used a less strict way of calculation. Finally, we had to exclude from the analyses all sound files with multiple temporal foci (e.g., both past- and future-oriented utterances in one sound file), as our unit of analysis was the “sound file.” In Study 2, we used the same strategy for consistency across studies, but also ran additional analyses with all sound files without any exclusions.

## Study 2

In Study 2, we validated our coding scheme with two new samples from a different country. We observed healthy young and older adults in Switzerland for 4 days. Our goal was to examine whether the coding scheme used in Study 1 would lead to similar results with participants (1) from different age groups, (2) from Switzerland who speak a different language (i.e., Swiss German), and (3) who were observed for a longer period of time that also included weekdays.

We expected our finding on conversational time travel to be replicated: Past-oriented utterances should outnumber future-oriented utterances independent of age group, country of origin (and language) and sampling rate of EAR. In terms of age effects, Park et al. (2016) found that across all age groups (between ages 13–48), the rank order of past, present and future orientation remained the same: Present-oriented social media messages were the most frequent, followed by past-oriented and then future-oriented messages (the difference between past and future was very small). However, there were some differences in the relative proportion of each orientation across age. We expected to find similar results, with a retrospective bias in conversational time travel for all age groups. There are no cross-cultural studies on mental time travel, but we did not anticipate country of origin to have a major impact, as mental time travel is a universal human ability (Suddendorf and Corballis, 2007). In terms of sampling rate effects, Gardner and Ascoli (2015) tested different sampling intervals in their experience-sampling study (e.g., weekend versus weekday, early in day versus late in day) and found no significant effect on the prevalence of past- versus future-oriented thoughts. We also did not expect sampling rate to affect our results.

### Materials and Methods

#### Sample

Our sample of real-life situations included 9,827 sound snippets collected from 33 young adults (19–31 years, *M* = 23.76, *SD* = 3.03; 10 men, 23 women) and 13,276 sound snippets collected from 48 healthy older adults (62–83 years, *M* = 70.54, *SD* = 4.65; 22 men, 26 women). Participants were recruited via the participant pool of the Gerontopsychology Lab at the University of Zurich, via flyers in university buildings and advertisements in a local newspaper, and through snowball sampling used by a research assistant. All participants lived in Switzerland and spoke Swiss German. Young participants were mostly university students, with number of years of education ranging between three and 17 (*M* = 12.18, *SD* = 2.32). Sixty-nine percent of them were single, whereas 31% were in a long-term relationship.

Older participants were healthy with no record of neurological or psychiatric illness and lived independently. 60% were married (with 4 couples within the sample) and 40% were divorced, widowed or single. Forty-six percent lived alone, 44% lived with one person in the same household and the remaining 10% lived with more than one person in the same household. Number of years of education ranged between seven and 25 (*M* = 10.55, *SD* = 3.02). An inclusion criterion for the study was a minimum score of 27 on the Mini Mental State Examination (MMSE; Folstein et al., 1975) and all participants were above this cut-off score (*M* = 29.2, *SD* = 0.84). Older participants were compensated with 50 Swiss Francs, whereas young participants could choose between 50 Swiss Francs and research credits.

#### Procedure

Participants met the researchers for an introduction session, after which data collection with the EAR started. Data collection spanned four consecutive days. Finally, participants met with the researchers again for a feedback session.

##### Introductory laboratory session

Participants came to the Psychology Institute for the first session, typically held on a Wednesday or a Friday afternoon. Six older participants were visited at home for their convenience. Participants were given instructions on the study, asked to sign an informed consent form and to complete questionnaires including demographic and psychological measures. All questionnaires were administered in a group setting except for the MMSE which was administered privately. Next, participants received their assigned iPhone with its protective case and charging cable. They were asked to think of the iPhone as a “recorder,” as it was set to “Airplane mode” and locked with only the EAR application on. They were reminded to carry the iPhone as much as possible over the next 4 days during their waking hours. They were told that the EAR would record 30 s of ambient sounds at a time, and that they would not be aware of when the EAR was recording, so that they could continue their normal lives. They were also informed that they would have the opportunity to review and delete any sound files at the end of the study, before anyone listened to them.

##### EAR data collection

Data collection spanned 2 weekdays and 1 weekend in counterbalanced order: 46 participants started data collection on a weekday (Thu, Fri, Sat, Sun) and 35 participants started on a weekend (Sat, Sun, Mon, Tue). Over these 4 days, participants carried the iPhone either clipped to their waistline or in their pockets. They did not have to do anything with the iPhone other than carrying it and charging it every night. Participants also filled out a short diary each day, in which they reported their main activities throughout the day and indicated when they were and were not carrying the EAR and whether they preferred any sound files from a certain time slot to be deleted due to privacy reasons.

##### Final laboratory session

After 4 days of data collection, typically on a Monday or a Wednesday, participants returned to the Psychology Institute or were revisited at home. The researcher collected the iPhones, the charging cables and the diaries, and administered a second questionnaire packet. The packet included psychological measures, as well as a questionnaire in which participants evaluated their experience with carrying the iPhone (e.g., degree to which they and others were aware of the EAR, degree to which carrying the iPhone changed their behavior). While participants filled out the questionnaires, the researcher downloaded the recorded sound files onto a lab computer and checked whether there were any problematic files. As participants had the right to listen to their sound files, the researcher burned a CD that included all of their files. Participants could either review their sound files in the lab and permanently delete any files they wished to have deleted, or they could receive the CD to review at home and inform the researcher within 10 days about any deletion requests. In the young group, 9 participants deleted between 1 and 40 sound files, 87 in total. In the old group, 6 participants deleted between 2 and 25 sound files, 46 in total.

#### Measures

Each participant was provided with an iPhone 4S which had the EAR application installed (version 2.3.0). The app was programmed to record 30-s sound snippets every 15 min, but with 100% randomization so that recordings were randomly distributed throughout the day (72 per day). The app was active for four consecutive days, 18 h per day with a blackout period between midnight and 6 AM each day (72 days × 4 days = 288 recordings per participant). In total, only 2.5 % of the participant’s day (i.e., 36 min) was recorded, which kept possible intrusions into participants‘ private lives on a minimal level. The iPhone was set to “Airplane mode” and locked with a screen-lock password, therefore the participants could not access the EAR settings or use the phone for other purposes. Participants were instructed to charge the iPhone overnight, but as a reminder the phone calendar was programmed to automatically beep every evening at 9 PM.

##### EAR-Derived measures: coding of sound files

Similar to Study 1, each sound file was coded in terms of whether the participant was talking or not, and if talking, for the temporal focus of the participants’ utterances (Table 1). All coding categories were dichotomous (1 versus 0) indicating presence or absence of a category. Similar to Study 1, we also had the TIME variable (1 = personal past, 2 = others’ past, 3 = present, 4 = personal future, 5 = others’ future, 6 = time-independent). In Study 2, we improved this variable and made it much more fine-grained by adding *all* possible combinations of temporal foci (1–2 = personal past and others’ past, 1–3 = personal past and present, 1-2-3 = personal past, others’ past and present, and so on). Furthermore, we created a new DOMINANT TIME variable, which categorized every sound file that includes more than one temporal focus in terms of which temporal focus is best represented (Table 2). This new variable allowed every sound file (every unit of analysis) to have a single temporal focus, which allowed us to include all sound files in our analyses.

All sound files were listened to and coded by two trained coders. Similar to Study 1, when we used the strict strategy of calculating inter-rater reliability using the TIME variable, reliability was 62.12%. However, in this study, we also used a lenient strategy: We calculated inter-rater reliability separately for each temporal focus which led to higher agreement between the coders (Personal past = 90.88%, Others’ past = 94.77%, Present = 77.43%, Personal future = 94.87%, Others’ future = 96.92%, Time-independent = 80.83%). All sound files that showed a disagreement between the two coders were re-listened to and the disagreement was resolved through discussion among the two coders.

### Results

#### Preliminary Analyses

In the young sample, a total of 2,087 sound files included participant speech (21%). We were unable to code for temporal focus in 167 sound files (8%) due to the brevity or vagueness of speech. Of the remaining 1,920 sound files, 255 included more than one temporal focus (13%). The remaining 1,665 sound files included only a single temporal focus. The older sample had 2,590 sound files with (21%) participant speech. Out of these, temporal focus was unidentifiable in 336 files (13%). Of the remaining 2,254 files, 315 included more than one temporal focus (14%). The remaining 1,939 files included only a single temporal focus.

#### Major Analyses

The goal of Study 2 was to use our validated coding scheme to examine the prevalence of past- versus future-oriented utterances and to replicate Study 1 results (i.e., retrospective bias in conversational time travel). Analyses were conducted in two ways: (1) Similar to Study 1, with sound files that included only a single temporal focus, and (2) with all sound files that included both single and multiple temporal foci, by using the new DOMINANT TIME variable.

##### Young adults

**FIGURE 2:**
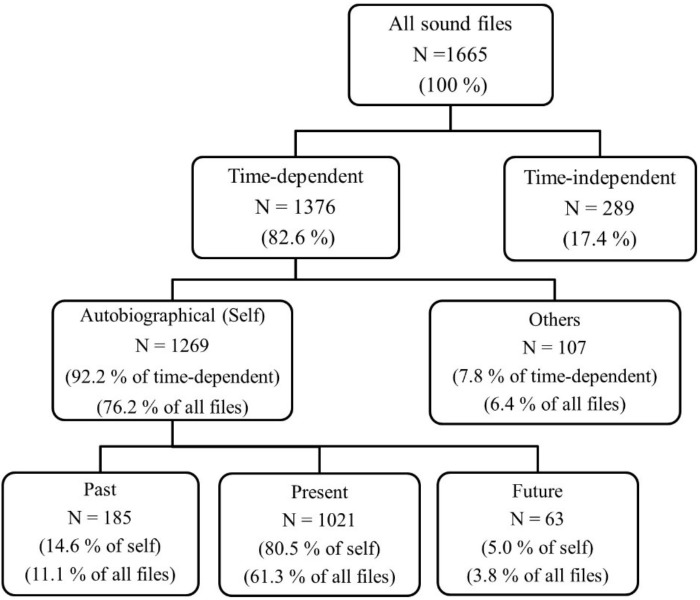
Study 2, Young adults: frequencies and percentages for each temporal category. All sound files (100%) refers to all sound files that include speech with no technical problems.

(1) Similar to Study 1, we first ran analyses with only the sound files that included a single temporal focus. We found exactly the same percentages for the young adults’ time-dependent versus time-independent, and self-related versus others-related sound files (Figure 2, first three rows). That is, similar to Study 1 participants, young Swiss adults referred to time in 82.6% of their sound files and talked about semantic memory or personal comments in 17.4%. Again similar to Study 1, out of the time-dependent sound files, 92% were about the self and 8% were about others. Utterances about others were further divided into others’ past (79.4%) and others’ future (20.6%). Sound files that included self-related utterances were further divided into past (14.6%), present (80.5%) and future categories (5%) to present mental time travel (Figure 2, bottom row). This is where young adults diverged slightly from Study 1 participants. They talked about their personal past in 11% of all their sound files and about their future in about 4% (14.9% of total conversational time travel in their sound files). We conducted a repeated-measures ANOVA to compare the aggregated person-level amount of past-oriented utterances with future-oriented utterances and found that young adults talked significantly more about their past (*M* = 5.58, *SD* = 4.21) than their future (*M* = 1.85, *SD* = 1.58), *F*(2,31) = 58.16, *p* < 0.001, η_ρ_^2^ **=** 0.079.

Similar to Study 1 participants, young adults’ utterances about others were divided into others’ past (78.5%) and others’ future (21.5%). Once more, present was the most frequent category (61%, *M* = 30.97, *SD* = 17.30), significantly more frequent than both past- and future-oriented utterances, pairwise comparisons: *t* ranges -8.71 to 10.09, *p*s < .001. As expected, the retrospective bias in conversational time travel was replicated with the same rank order of present, past and future orientation (Park et al., 2016).

(2) Next, we calculated percentages with the new DOMINANT TIME variable and used all sound files, including those with multiple temporal foci. We found almost the same percentages as in Figure 2 (See Supplementary Figure 1A). The only difference was that the percentages slightly increased for conversational time travel: Personal past was the dominant temporal focus in 12.7% of the sound files, whereas personal future was the dominant temporal focus in only 5.5% of the sound files (as opposed to 11.1% versus 3.8% in Figure 2). We conducted a repeated-measures ANOVA and found that young adults talked significantly more about their past (*M* = 7.36, *SD* = 4.66) than their future (*M* = 3.21, *SD* = 2.56), *F*(2,31) = 55.07, *p* < 0.001, η_ρ_^2^ **=** 0.078. In summary, this shows that the two different ways of calculating percentages led to similar results.

**FIGURE 3:**
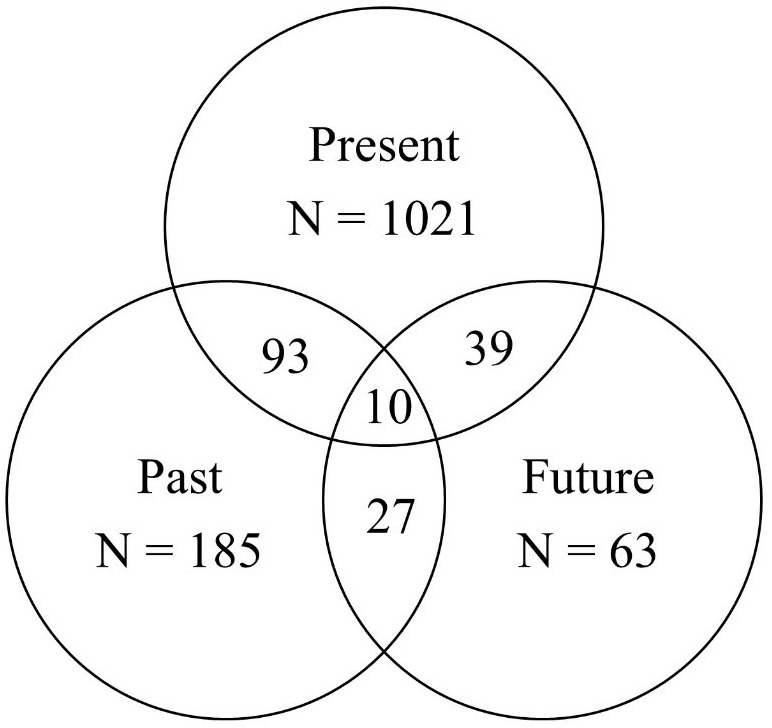
Study 2, Young adults: basic Venn diagrams for the frequencies of autobiographical, time-dependent utterances.

In addition, we created Venn diagrams of autobiographical, time-dependent utterances to take a more detailed and closer look at conversational time travel frequencies. As depicted in Figure 3, young adults referred to their personal past (*N* = 315) much more than their personal future (*N* = 139).

##### Older adults

**FIGURE 4:**
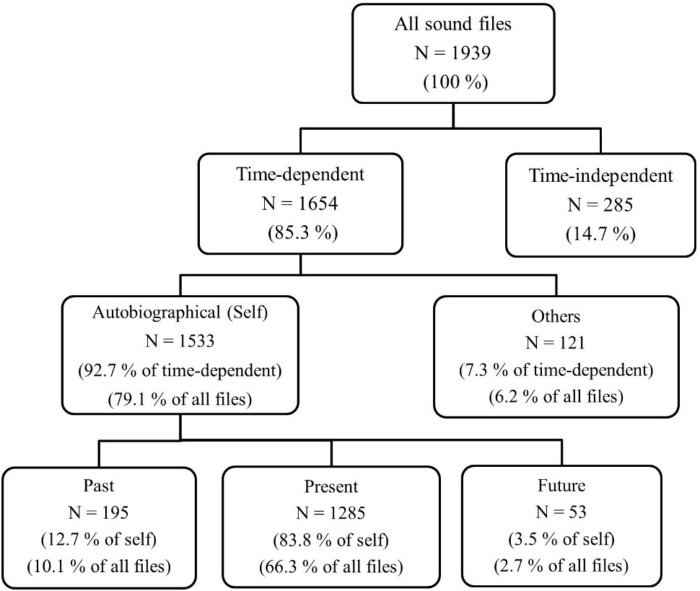
Study 2, Older adults: frequencies and percentages for each temporal category. All sound files (100%) refers to all sound files that include speech with no technical problems.

(1) Similar to Study 1, we first ran analyses with only the sound files that included a single temporal focus. We found that older adults had very similar percentages to the young (Figure 4). Ten percent of older adults’ sound files were about the personal past, whereas only 2.7% were about the personal future (Figure 4, bottom row). We were unable to conduct a repeated-measures ANOVA due to the non-normal distributions of the difference scores of each temporal focus (i.e., past-present, present-future, future-past) as shown by Shapiro–Wilk normality tests, *W*s ranged between 0.89 and 0.95, *p*s < 0.05. Therefore, we ran the non-parametric equivalent, Wilcoxon signed-rank test. We found that older adults talked significantly more about their past (*Mdn* = 3.00) than their future (*Mdn* = 1.00), *V* = 61.5, *p* < 0.001, *r* = -0.48.

Similar to Study 1, present (66%, *M* = 26.85, *SD* = 17.04) was significantly more frequent than both past- and future-oriented utterances, V ranges 1173–1176, *p*s < 0.001. As expected, the retrospective bias in conversational time travel was replicated with the same rank order of present, past and future orientation (Park et al., 2016).

We also examined the interaction between age group (young, old) and temporal focus (past, present, future), which was non-significant, *F*(2,78) = 0.58, *p* = 0.56. This suggests that the rank order of present, past and future orientation was similar across the two age groups. Finally, we calculated these percentages separately for weekdays and weekends. For both age groups, the percentages are highly similar to the original percentages (See Supplementary Table 1). Therefore, we can conclude that the retrospective bias holds similarly in both weekdays and weekends.

(2) Next, we calculated percentages with the new DOMINANT TIME variable and used all sound files, including those with multiple temporal foci. We found almost the same percentages as in Figure 4 (See Supplementary Figure 1B). Similar to young adults’ results, the only difference was that the percentages slightly increased for conversational time travel: Personal past was the dominant temporal focus in 11.2% of the sound files, whereas personal future was the dominant temporal focus in only 4% of the sound files (as opposed to 10.1% versus 2.7% in Figure 2). We conducted a repeated-measures ANOVA and found that older adults talked significantly more about their past (*M* = 5.25, *SD* = 5.25) than their future (*M* = 1.88, *SD* = 1.42), *F*(2,46) = 53.85, *p* < 0.001, η_ρ_^2^ **=** 0.070. Thus, we can conclude that, for both young and older adults, the two different ways of calculating percentages led to highly similar results.

**FIGURE 5:**
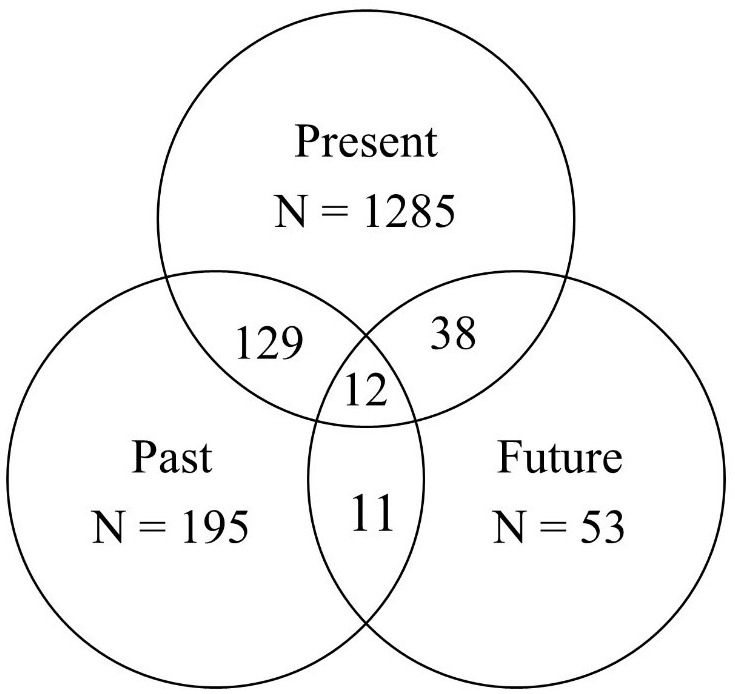
Study 2, Older adults: basic Venn diagrams for the frequencies of autobiographical, time-dependent utterances.

Finally, we created Venn diagrams of older adults’ autobiographical, time-dependent utterances. As depicted in Figure 5, older adults referred to their personal past (*N* = 347) much more than their future (*N* = 114). In conclusion, the retrospective bias was confirmed with Swiss older adults, similar to Swiss young adults and American adults.

## Discussion

This study aimed to validate our coding scheme and to replicate Study 1 results. Hence, it built on Study 1 in three ways. First, we recruited both men and women, and obtained more gender-balanced samples. This helped us to validate our coding scheme with different samples and to test whether the retrospective bias observed in Study 1 (i.e., with mostly middle-aged men) would generalize to these samples. Indeed, we showed that the results were highly similar across American and Swiss adults from different age groups.

Second, we used different EAR sampling rates across the two studies and tested whether this would have an impact on the results. The duration (50 versus 30 s) and the distribution of recordings (every 9 min versus random) did not influence the results. The advantage of Study 2 was that we collected data across 4 days (i.e., 1 weekend similar to Study 1 plus 2 weekdays). We found no difference between the weekend and weekdays in terms of the prevalence of past- versus future-oriented utterances. This shows that people are more likely to talk about their past than their future on both weekends and weekdays.

Third, we built on Study 1 with new analyses that did not exclude sound files with multiple temporal foci. That is, we conducted analyses with (1) sound files that included only a single temporal focus, and (2) all sound files with single and multiple temporal foci (by using the new DOMINANT TIME variable). The two sets of analyses revealed very similar percentages for both young and older adults. In addition, analyses of variance showed the same results with past-oriented utterances being significantly more frequent than future-oriented utterances. This is an indicator of the robustness of our findings. In sum, for young and older adults, 10.1–12.7% of their sound files were about their personal past, whereas only 2.7–5.5% of their files were about their personal future. The retrospective bias in conversational time travel was replicated with the same rank order of present, past and future orientation as in Study 1.

## General Discussion

This work is the first to examine mental time travel reflected in everyday conversations and to introduce the term “conversational time travel.” It is also the first to examine mental time travel using a naturalistic observation method. We used the EAR to observe the overt behavior of talking, rather than focusing on private thinking as has been done in previous work (e.g., Gardner et al., 2012). Much of human behavior and cognition occurs in social settings (Mehl and Pennebaker, 2003), therefore we aimed to investigate whether the prevalence of conversational time travel is different from private mental time travel. Using the EAR also allowed us to evade possible limitations of the self-report method (e.g., memory errors, response biases, participant burden; Scollon et al., 2003), and to develop an ecological, objective and standard way of assessing conversational time travel. Furthermore, it allowed us to sample both across real-life situations and across individuals to maximize the diversity of situations to establish ecological validity.

### Validation of the Coding Scheme

The first goal of this research was to develop and validate a coding scheme for conversational time travel. We validated our scheme with a text analysis program (i.e., LIWC). We showed that utterances that we manually coded as time-dependent included more verbs with past and future tense than utterances coded as time-independent (i.e., semantic memory and general comments). Second, utterances that we coded as autobiographical were more about the self with first person pronouns (“I,” “we,” and “us”), whereas others-related utterances included more second and third person pronouns. Finally, we validated our mental time travel categories: Utterances coded as personal past included the highest number of verbs with past tense; utterances coded as personal future included the highest number of verbs with future tense and present-oriented utterances included the highest number of verbs with present tense. In sum, we succeeded in validating the whole coding scheme with LIWC.

Next, we validated our coding scheme with participants from (1) different age groups (i.e., young, middle-aged, old), (2) two countries that speak different languages (i.e., English and Swiss German), and (3) different EAR sampling designs. Across these different person samples, we acquired the same inter-rater reliability (with strict strategy in Study 1: 64.12% and Study 2: 62.12%). In Study 2, we also used a lenient strategy, which led to very high agreement between coders for conversational time travel (personal past = 90.88%, personal future = 94.87%). This suggests that our coding scheme is robust and reliable across different persons and situation samples. Furthermore, we achieved highly similar results across our person samples, which indicates that our results did not vary due to inconsistencies in coding across studies.

### Retrospective Bias in Conversational Time Travel

The second goal of the current research was to compare the prevalence of past- and future-oriented utterances across young, middle-aged and older adults in the United States and Switzerland. Our results first revealed that individuals mostly produced time-dependent utterances in everyday life conversations (82.5–85.3% of all sound files across all samples). Semantic information and general comments about the world occurred in only 14.7–17.5% of the recorded situations across samples (Suddendorf et al., 2009). This suggests that time mattered greatly for everyone while communicating with others. This is not surprising, as time is an inescapable aspect of our life-space (Lewin, 1939) that shapes our lives, including our social interactions (Webster, 2011).

Second, we found that individuals talked in a self-referential way across most of the situations: 76.2–79.1% of all sound files included autobiographical utterances across person samples. More specifically, 93% of time-dependent sound files included autobiographical utterances across samples. These percentages show that the majority of participants’ utterances were both time-dependent and autobiographical indicating that people tend to talk mostly about “self in time.” In contrast, participants talked about other people in only 5.8–6.4% of the sound files across all person samples. This suggests that vicarious memories (Pillemer et al., 2015) and vicarious future-oriented utterances (e.g., Grysman et al., 2013) occur quite rarely in conversations. Bryant et al. (2013), using signal-contingent sampling, also found that individuals experienced a higher number of self-related thoughts than others-related thoughts. Future research should further investigate the significance and functions of vicarious thoughts and utterances about others.

Finally, we examined mental time travel as reflected in participants’ autobiographical utterances and found that 10.1–13.6% of their sound files were about the personal past, whereas 2.7–7.2% were about the personal future. That is, individuals across samples talked about their personal past two to three times as much as their personal future. This is in line with our expectation of a retrospective bias in the social setting of conversations, and in contrast to previous work on private thoughts: While thinking, individuals seem to focus more on their future than their past (e.g., Song and Wang, 2012). Future-oriented thinking serves directive functions such as planning, decision making, problem solving, goal intention and goal achievement (e.g., Szpunar, 2010; D’Argembeau et al., 2011; Schacter et al., 2017). For example, Barsics et al. (2016) examined the functions of emotional future-oriented thoughts and found that participants self-reported four major functions: to plan actions, form intentions (i.e., to set goals), make decisions, and regulate emotions. Twenty percent of emotional future-oriented thoughts were rated as not functional and 5% were reported to involve other kinds of functions, such as daydreaming. Cole and Berntsen (2016) showed that participants’ future representations were more frequently related to their goals (i.e., current concerns) than their autobiographical memories. Furthermore, future-oriented mind wandering is found to be more self-related and directive than past- and present-oriented mind wandering (Baird et al., 2011; Stawarczyk et al., 2011). All of these results show that future-oriented thoughts do not tend to serve social functions. Therefore, they are not highly frequent or relevant in social interactions. They are more useful when people are thinking alone, as directive functions seem to be inherently private (Kulkofsky et al., 2010; O’Rourke et al., 2017).

In contrast, past research on autobiographical memories underlines significant social functions of memories showing that people recall their past to provide material for conversation (Hyman and Faries, 1992; Pasupathi et al., 2002), to create/enhance intimacy in relationships (Alea and Bluck, 2007), to elicit empathy for others (Bluck et al., 2013) and to teach and inform others (O’Rourke et al., 2017). For example, Demiray et al. (2017) examined how and why older adults reminisced about their past in real-life conversations. They coded participants’ utterances that included reminiscence in terms of their functions and found that reminiscence served mainly social functions (i.e., conversation, teaching) and did not serve any directive functions (e.g., problem solving, death preparation). Therefore, it is not surprising for us to have found a retrospective bias in conversational time travel: Social settings and cues seem to trigger the recall of autobiographical memories.

Indeed, Vannucci et al. (2017) showed, in spite of the widely observed prospective bias in mind wandering, that using external verbal cues in the experimental task changed the nature of mind wandering: They found that task-irrelevant verbal cues directed the temporal orientation of mind wandering toward the past. In the Verbal-cues group, 44.5% of mind wandering episodes were categorized as memories and 18.3% as future-oriented thoughts. In contrast, in the No-cues group, 28.3% were classified as memories, whereas 38.7% as future-oriented thoughts. Furthermore, Mazzoni et al. (2014) found that more involuntary memories were elicited when verbal cues rather than pictorial cues were presented, whereas there was no difference between the effects of verbal and pictorial cues on other spontaneous (and non-memory) thoughts. More generally, it has been shown that external/environmental cues primarily trigger past-oriented thoughts (Berntsen and Jacobsen, 2008; Maillet and Schacter, 2016a). All of these findings suggest that spontaneous past-oriented thinking is affected by external cues (rather than internal cues, such as mood), and especially by verbal cues (Plimpton et al., 2015). This link between environmental cues and past-oriented thinking may be an important adaptive mechanism that allows individuals to relate the current situation to similar events experienced in the past, which might support adaptive behavior (Maillet and Schacter, 2016b). Conversations are strong verbal cues, which might be one factor underlying the retrospective bias we discovered in conversational time travel. In contrast, spontaneous future thinking is mainly related to and triggered by private concerns, being less dependent on external stimuli (Klinger, 2013; Cole and Berntsen, 2016).

In sum, the retrospective bias in conversational time travel seems to be a universal phenomenon across situations and persons (e.g., Suddendorf and Corballis, 2007; Suddendorf et al., 2009), as all of our samples revealed very similar percentages. Although coming from different countries, age groups and research designs, all samples focused on their past much more than their future during conversations. Across all age groups, the retrospective bias in conversational time travel was replicated with the same rank order of present, past and future orientation (Park et al., 2016). Past work shows that the frequency of recalling the personal past does not vary by age (Webster, 1999; Pasupathi and Carstensen, 2003; Gardner and Ascoli, 2015). Our results on talking behavior are in line with this finding on thinking. Gardner and Ascoli (2015) found that older adults thought about their future twice (21%) as much as young adults (10%). We found, however, that older adults were quite similar to younger individuals in terms of the frequency of talking about the personal future. These findings contradict with the socioemotional selectivity theory (Carstensen, 1992), which states that older adults have a less positive and open-ended future time perspective than young adults (Demiray and Bluck, 2014). This suggests that one’s subjective and global perspective of their future may not be associated with how much they think or talk about their future in everyday life. Thus, future studies could examine conversational time travel via both subjective self-report and objective observation.

### Methodological Issues in Measuring the Prevalence of Mental Time Travel

Previous studies measuring the incidence of subjective thoughts have typically used the experience-sampling method. However, event-contingent sampling (i.e., diary method, Berntsen, 2007) has some limitations. For example, in the case of examining involuntary autobiographical memories (spontaneously popping in mind), the method requires that the participant first understands what qualifies as an involuntary memory. Next, when a memory comes into awareness, the participant must retrospectively identify the experience as “memory retrieval” (Note that some may not be sufficiently activated to pass the awareness threshold; Barzykowski and Staugaard, 2017; Vannucci et al., 2017). Then, the participant must decide that the recollection is something worth reporting in the diary. All of these requirements create a cognitive burden to the participants and the risk that many memories may go undetected or ignored due to demotivation or exhaustion (Hintzman, 2011; Vannucci et al., 2014; Barsics et al., 2016). Finally, informing participants about the phenomenon of interest may bias them toward thinking more about the past or toward voluntarily monitoring their thoughts (D’Argembeau et al., 2011; Barzykowski and Niedźwieńska, 2016). Indeed, Barsics et al. (2016) showed, in their diary study, that participants reported having experienced more thoughts than usual because they were requested to record them. Due to these limitations, we do not think that the diary method is the ideal method to examine the natural frequency of past- versus future-oriented thoughts.

Signal-contingent sampling is advantageous over the diary method in that it allows for a random sampling of experiences and avoids expectancy effects (Scollon et al., 2003). It is considered the gold standard for the assessment of cognitive or behavioral processes in everyday life, since recall biases and heuristic biases are minimized (Shiffman et al., 2008). However, participant burden is still an issue and assessments may be reactive. Some participants have reported that the signals interrupted their thoughts, which might have led to confusion and possible misratings in the questionnaire (Bryant et al., 2013). Similar to event-contingent sampling, making participants aware of study aims might affect their responses. For example, asking participants to perform a mental check at each signal on whether they had been thinking about a memory or not (Gardner et al., 2012) might alter their experience. Indeed, research shows that participants who were asked to selectively report memories did this to a greater extent than participants asked to report any type of thought (Vannucci et al., 2014; Barzykowski and Niedźwieńska, 2016; Barzykowski and Staugaard, 2017).

Therefore, automatized and unobtrusive methods that do not reveal study aims and that minimize participant burden, such as the EAR, are advantageous while examining observable phenomena that do not require self-report. They maximize ecological validity, as huge amounts of data can be collected without experimenter or participant burden, and contextual influences on experience can be detected (Mehl, 2017). However, although the EAR is an ideal method to examine conversations, it cannot be used to assess thoughts. Thus, signal-contingent sampling method and the EAR should be combined as two strong ecological methodologies with different advantages (Mehl et al., 2012). This should create a uniquely powerful way of studying thought processes in natural habitats with the fine-grained multi-method approach.

### Limitations

One limitation of the current study is its sole dependence on the coding and analysis of overt speech data. A multi-method approach that also collects self-reports from participants could inform us about what is happening in participants’ minds. Experience-sampling method (merged with the EAR) could help us understand how and why individuals are engaging in conversational time travel in certain situations. This method would allow us to examine both thinking and talking behaviors within the same study and to compare how these two modalities shape mental time travel. A strength of our study, however, is that it demonstrates that meaningful information can be derived from the observation of real-life verbal activities. This may allow us to include (older) persons in research who may feel overly burdened or are unable to reliably self-report information and are, so far, excluded from research.

One limitation of our coding scheme is that it does not differentiate between self-related versus others-related time-independent utterances. This distinction was not within the scope of the current study, however, future research could enhance the coding scheme with two separate dimensions for temporal focus (e.g., past, present, future, none) and subject (e.g., self, others, none).

Another limitation is that we have taken a between-persons approach and neglected the within-person dynamics of conversational time travel. The retrospective bias in conversational time travel seems to be a universal phenomenon, however, there are individual differences in how much people talk about their past or future (Demiray et al., 2017). Future work should focus on within-person variability in mental/conversational time travel across situations and examine the impact of context on the frequency, characteristics and functions of thinking and talking about the past versus future. For example, Study 2 did not include middle-aged adults, who are active in the workforce and who may be using work-related language throughout the weekdays that is mostly time-independent (e.g., semantic information). Such contextual effects (e.g., conversation partners; Demiray et al., 2017) and the topic of conversations should be examined in future research. Finally, our older sample included 4 couples, whose data may be dependent on each other. However, it is highly unlikely that duplicate sampling of the same 30-s sound-snippets occurred, as recordings were 100% randomly distributed.

## Conclusion

The current research has introduced the term “conversational time travel” and examined its prevalence in everyday life. It seems that individuals, across widely varying real-life situations, talk two to three times more about their personal past than their future. This retrospective bias in conversational time travel highlights the social functions of recalling and sharing the personal past with others. Talking about past experiences seems to be an adaptive behavior that helps us to connect with others and to survive in this social world.

## Ethics Statement

Study 1 has been approved by the Institutional Review Board of the University of Arizona, and Study 2 was approved by the Ethics Committee of the University of Zurich. All participants, first, gave written informed consent in accordance with the Declaration of Helsinki.

## Author Contributions

BD developed the research concept and the research design. MRM conducted Study 1 for a larger project with prior students. BD conducted Study 2, collected the data, performed the data coding, and analyses and drafted the manuscript. BD and MRM worked together on the interpretation of results and on framing the manuscript. MM provided the critical revisions on the manuscript.

## Conflict of Interest Statement

The authors declare that the research was conducted in the absence of any commercial or financial relationships that could be construed as a potential conflict of interest.
