# Supplementary material for: Conversational Time Travel: Evidence of a Retrospective Bias in Real Life Conversations
Source: Front Psychol. 2018 Nov 13;9:2160. doi: 10.3389/fpsyg.2018.02160 (PMC6243041; doi:10.3389/fpsyg.2018.02160)
Supplement: Supplementary file 1 [file Data_Sheet_1.docx]

Supplementary Material

**Conversational Time Travel:**

**Evidence of a Retrospective Bias in Real Life Conversations**

Burcu Demiray*, Matthias R. Mehl, Mike Martin

*** Correspondence:** Burcu Demiray: b.demiray@psychologie.uzh.ch

**Supplementary Table 1**

*Percentages of Past-, Present- And Future-Oriented Utterances for Young Versus Older Adults On Weekdays and Weekends*

|  | Past | Present | Future |
| --- | --- | --- | --- |
| Young, Weekdays | 11.88 | 58.22 | 4.44 |
| Young, Weekend | 10.33 | 64.58 | 3.20 |
| Old, Weekdays | 8.98 | 66.99 | 3.13 |
| Old, Weekend | 11.24 | 65.39 | 2.30 |

a. Young adults b. Older adults

| 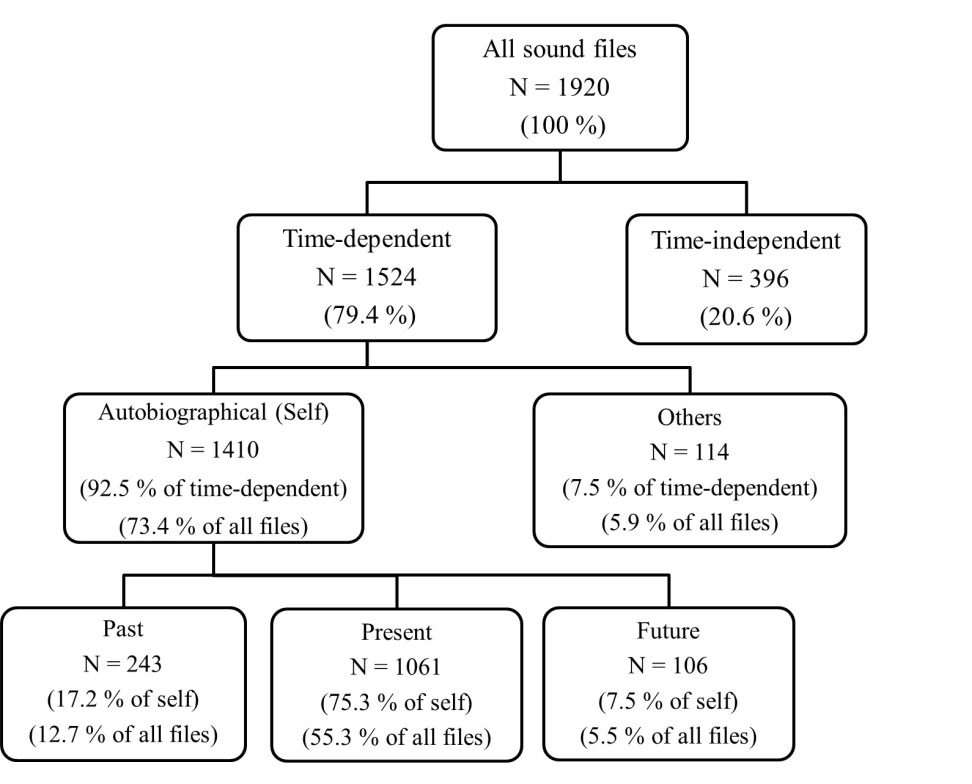 | 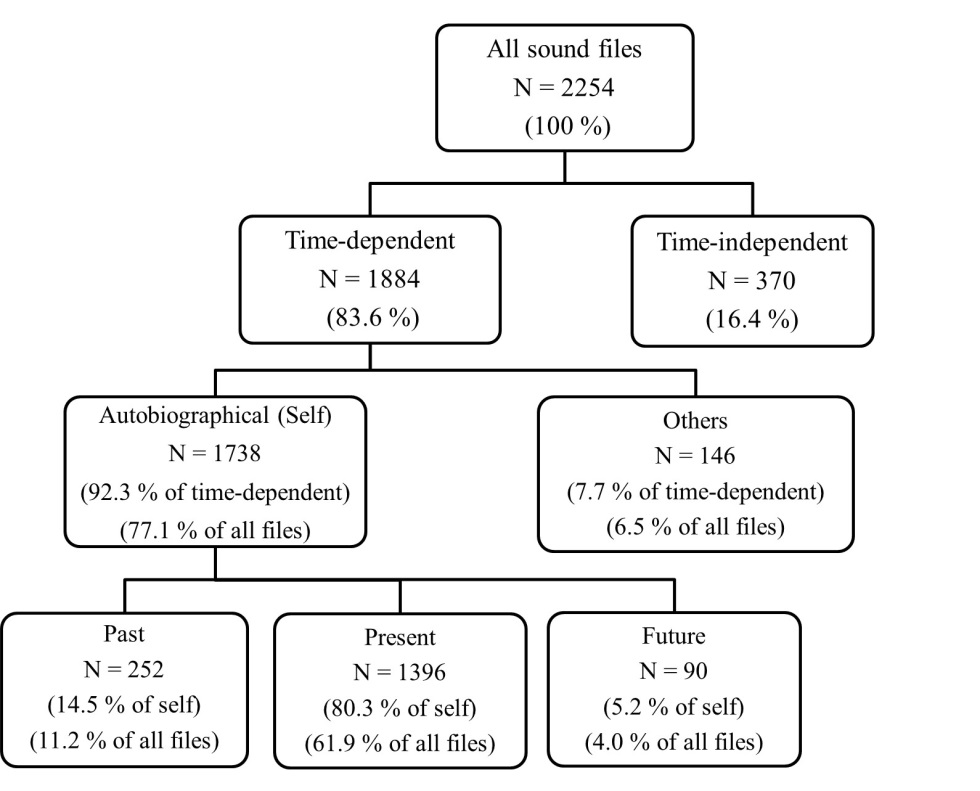 |
| --- | --- |

**Supplementary Figure 1.** Study 2: Frequencies and percentages for each temporal category calculated by using the DOMINANT TIME variable. All sound files (100%) refers to all sound files that include speech with no technical problems.
